# “A Step and a Ceiling”: mechanical properties of Ca^2+^ spark vasoregulation in resistance arteries by pressure‐induced oxidative activation of PKG

**DOI:** 10.14814/phy2.14260

**Published:** 2019-11-28

**Authors:** Viktoria Csato, Sharifah Z. S. A. Kadir, Kaivan Khavandi, Hayley Bennett, Sarah Sugden, Alison M. Gurney, Harry T. Pritchard, David Hill‐Eubanks, Philip Eaton, Mark T. Nelson, Adam S. Greenstein

**Affiliations:** ^1^ Division of Cardiovascular Sciences Faculty of Biology, Medicine and Health University of Manchester Health Innovation Manchester Network Manchester United Kingdom; ^2^ Division of Clinical Physiology Institute of Cardiology Research Centre for Molecular Medicine Faculty of Medicine University of Debrecen Debrecen Hungary; ^3^ Department of Pharmacology Faculty of Medicine University of Malaya Kuala Lumpur Malaysia; ^4^ Department of Pharmacology University of Vermont Burlington Vermont; ^5^ Centre for Clinical Pharmacology William Harvey Research Institute Queen Mary University of London London United Kingdom; ^6^ Present address: Centre for Clinical Pharmacology William Harvey Research Institute Queen Mary University of London London United Kingdom

**Keywords:** Ca^2+^ spark, oxidant signaling, pressure‐ induced constriction, protein kinase G, vascular smooth muscle

## Abstract

We investigated the biomechanical relationship between intraluminal pressure within small mesenteric resistance arteries, oxidant activation of PKG, Ca^2+^ sparks, and BK channel vasoregulation. Mesenteric resistance arteries from wild type (WT) and genetically modified mice with PKG resistance to oxidative activation were studied using wire and pressure myography. Ca^2+^ sparks and Ca^2+^ transients within vascular smooth muscle cells of intact arteries were characterized using high‐speed confocal microscopy of intact arteries. Arteries were studied under conditions of varying intraluminal pressure and oxidation. Intraluminal pressure specifically, rather than the generic stretch of the artery, was necessary to activate the oxidative pathway. We demonstrated a graded step activation profile for the generation of Ca^2+^ sparks and also a functional “ceiling” for this pressure –‐sensitive oxidative pathway. During steady state pressure ‐ induced constriction, any additional Ca^2+^ sensitive‐K^+^ channel functional availability was independent of oxidant activated PKG. There was an increase in the amplitude, but not the Area under the Curve (AUC) of the caffeine‐induced Ca^2+^ transient in pressurized arteries from mice with oxidant‐resistant PKG compared with wild type. Overall, we surmise that intraluminal pressure within resistance arteries controls Ca^2+^ spark vasoregulation through a tightly controlled pathway with a graded onset switch. The pathway, underpinned by oxidant activation of PKG, cannot be further boosted by additional pressure or oxidation once active. We propose that these restrictive characteristics of pressure‐induced Ca^2+^ spark vasoregulation confer stability for the artery in order to provide a constant flow independent of additional pressure fluctuations or exogenous oxidants.

## Abstract 2

Intraluminal pressure within resistance arteries triggers a binary activation of an oxidant‐driven pathway whereby protein kinase G regulates calcium spark vasoregulation. Once activated, the pathway cannot be boosted by additional pressure or oxidants and we propose this confers stability upon the pressure‐induced constriction of the artery.

## Graphical abstract

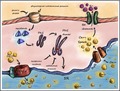

## Introduction

Coupling between sarcoplasmic reticulum (SR)‐derived Ca^2+^ sparks and plasmalemmal K^+^ channels of vascular smooth muscle cells (VSMC) is one of the most important vasodilatory pathways within small arteries (Nelson et al. 1995). Due to their spatiotemporally brief nature, Ca^2+^ sparks do not directly influence the overall cytoplasmic Ca^2+^ concentration within the myocyte. However, the local rise in Ca^2+^ from 200 nmol/L to 30 *µ*mol/L within the vicinity of the Ca^2+^ spark is sufficient to activate around 30 nearby plasmalemmal Ca^2+^‐activated K^+^ channels (BK channels) (Perez et al. 2001). The VSMC hyperpolarization resulting from this BK channel activation partially dilates the artery but in doing so blunts the inherent constriction triggered by intraluminal pressure (Nelson et al. 1995). Crucially, however, in addition to initiating pressure‐induced constriction, intraluminal pressure is also the principal driver for the formation of Ca^2+^ sparks within the artery (Knot and Nelson 1998; Jaggar 2001). This precise pressure‐ regulated control over small artery diameter is the basis for maintenance of microvascular diameter.

We recently reported that the mechanism by which intraluminal pressure initiates the vasodilatory Ca^2+^ spark pathway is through generation of oxidants which activate protein kinase G (PKG) (Khavandi et al. 2016). Thus activated, PKG regulates Ca^2+^ spark release from the SR. Our study exploited a genetically modified mouse whereby the cysteine residue at point 42 is switched to serine (hereafter termed PKG[C42S]^KI^) (Prysyazhna et al. 2012). Serine does not have a sulfur moiety and thus the capacity for oxidants to activate PKG through oxidative dimerization is prevented. In the PKG[C42S]^KI^ mouse mesenteric arteries, physiological levels of intraluminal pressure (80 mmHg) were not able to initiate vasodilatory Ca^2+^ sparks. Subsequently, the PKG[C42S]^KI^ arteries were more constricted and this was associated with hypertension.

Detection of this pathway: intraluminal pressure–oxidants–PKG–Ca^2+^ sparks–BK vasodilation, raised several questions regarding the biomechanics of Ca^2+^ spark vasoregulation such as the relationship between pressure‐related oxidants and Ca^2+^ spark frequency, the inflection point necessary to initiate the pathway, and the capacity of oxidants to further modulate PKG and thus Ca^2+^ spark vasoregulation. In this study, we addressed these questions by comparing diameter changes and Ca^2+^ signalling between WT and PKG[C42S]^KI^ arteries in response to a variety of physical and pharmacological stimuli. There was steep, almost digital activation of Ca^2+^ spark generation between 20 and 50 mmHg of intraluminal pressure and higher pressures had no further effect on Ca^2+^ spark frequency. Together with our previous results, our study contextualizes the functional contribution of pressure‐induced oxidative activation of PKG in the control of small artery diameter via Ca^2+^ spark vasoregulation.

## Materials and Methods

### Animal studies

Procedures were performed in accordance with the UK Home Office Guidance on the Operation of the Animals (Scientific Procedures) Act 1986 in United Kingdom and were approved by an institutional review committee. Mice constitutively expressing PKGI*α* Cys42Ser (referred to in the manuscript as PKG[C42S]^KI^) were generated on a pure C57BL/6j background by Taconic Artemis (Koln, Germany) as described previously (Prysyazhna et al. 2012). Colonies in Manchester were replenished annually by breeding PKG[C42S]^KI^ mice with commercially obtained C57Bl/6j wild‐type (WT) mice to generate heterozygous mice. From these heterozygotes, either WT or PKG[C42S]^KI^ colonies were generated and then maintained by breeding and genotyping. Age‐matched (12‐week‐old) male WT C57BL/6j and PKG[Cys42Ser]^KI^ mice were used in all studies. Mice had ad libitum access to standard chow and water and were kept in specific pathogen‐free conditions, under a 12‐h day/night cycle. Mice were euthanized by cervical dislocation. The mesenteric bed was removed and kept in an ice‐cold HEPES‐buffered physiological saline (HEPES‐PSS) with the following composition: 134 mmol/L NaCl, 6 mmol/L KCl, 1 mmol/L MgCl_2_, 2 mmol/L CaCl_2_, 7 mmol/L glucose, and 10 mmol/L HEPES, with pH adjusted to 7.4 with 1 mol/L NaOH. All chemicals were from Sigma (Dorset, UK), aside from the antibodies, which were obtained from Badrilla or the Jackson Immunoresearch Laboratories Inc.

### Genotyping

Mice were genotyped using the REDExtract‐N‐Amp Tissue PCR kit from Sigma‐Aldrich (Dorset, UK), as described by the manufacturer. Briefly, DNA was extracted from ear snip tissue (2–3 mm) by incubating it with Extraction Solution and Tissue Preparation Solution in a 4:1 ratio for 10 min at room temperature. The mixture was then transferred into a heating block and incubated at 95°C for 3 min, before digestion was stopped by adding the Neutralization solution. The extracted DNA was mixed with PCR ReadyMiX (Sigma‐Aldrich) and the PKG[C42S]KI‐specific primers, 5′‐cag ttt agg gac aga gtt gg‐3′ (forward) and 5′‐aac ctg ctt cat gcg caa gg‐3 (reverse), used at a final concentration of 0.4 *μ*mol/L.

### Immunoblotting

Mouse mesenteric arteries (either untreated circulations from mesenteric beds or grouped samples which had been wire mounted as described in section: “Wire myography”) were heated to 65°C for 10 min immediately prior to loading the gel. Nonreducing SDS‐polyacrylamide gel electrophoresis was carried out using a Bio‐Rad system (Hercules, CA). After electrophoresis, samples were transferred to polyvinylidene difluoride membranes using a Bio‐Rad semidry blotter. Membranes were blocked with 3.5% BSA in Tris‐buffered saline (TBS) containing 0.1% Tween (TBS‐T), overnight at 4°C. All proteins were imaged under UV excitation, using the BioRad Stain‐Free™ technology, to assess protein loading. Digitized immunoblots were collected using a Chemidoc system (BioRad) and analyzed with BioRad ImageLab software. For the PKG blot protocols, membranes were blocked with 10% nonfat dry milk in phosphate‐buffered saline containing 0.1% PBS‐T for 1 h at room temperature and then immunostained for PKG1*α* (ADI‐KAP‐K005‐F PKG; Enzo Life Sciences, Exeter, UK) as previously documented(Burgoyne et al. 2007; Prysyazhna et al. 2012). Monomeric (~75 kDa) and dimeric (~150 kDa) forms of PKG were quantified using ImageJ software.

### Pressure myography

Third‐order branches of mesenteric arteries (~130–150 *µ*m internal diameter) were dissected free of surrounding tissue. Some were snap frozen in liquid N_2_ and others mounted on borosilicate glass pipettes in an arteriograph chamber (Living Systems Instrumentation, St. Albans, VT and Ionoptix, MA). The proximal glass pipette was attached to a servo‐controlled pressure‐regulating device and the distal pipette was blocked. After checking for leaks, arteries were pressurized to 80 mmHg for approximately 45 min in physiological saline (PSS) with the following composition: 119 mmol/L NaCl, 4.7 mmol/L KCl, 1.2 mmol/L KH_2_PO_4_, 1.2 mmol/L MgCl_2_, 2 mmol/L CaCl_2_, 7 mmol/L glucose, 24 mmol/L NaHCO_3_, and 2.3 mmol/L EDTA. PSS was warmed to 37°C and continuously gassed with a 95% air and 5% CO_2_ mixture. Arteries exhibiting a leak were discarded and pharmacological protocols were performed only on arteries that exhibited pressure‐induced myogenic tone. Internal diameter was detected using a CCD camera and edge‐detection software. Pressurized arteries were exposed to circulating PSS and allowed to develop myogenic tone, defined as the difference between the vessel diameter in Ca^2+^‐containing and Ca^2+^‐free solutions, expressed as a percentage of the diameter in Ca^2+^‐free conditions. Dilation or constriction to drugs was expressed as the percent change in active tone. All drugs used were purchased from Sigma‐Aldrich.

### High‐speed spinning‐disc confocal microscopy

#### Measurement of Ca^2+^ sparks

Mesenteric arteries were placed in HEPES‐PSS containing 10 *µ*mol/L Fluo‐4‐AM and 0.05% pluronic acid for 45 min at room temperature, followed by a 15‐min wash in HEPES‐PSS. Arteries were subsequently mounted for pressure myography and pressurized to either 20, 30, 40, 50, 80, or 110 mmHg, in circulating PSS gassed with 95% air and 5% CO_2_. Ca^2+^ sparks were imaged with a confocal system (Revolution X system, Andor Technology, Belfast, UK) consisting of a Nikon upright microscope, 60× water‐dipping objective (NA 1.0) and electron‐multiplying Andor CCD camera. Images were acquired with Andor Revolution acquisition software at 55 frames/sec. Bound Ca^2+^ was detected by exciting at 488 nm and collecting emitted fluorescence using a 527.5/49 nm band‐pass filter. Video files of Ca^2+^ sparks were obtained from arteries before and after incubation with drugs. Ca^2+^ sparks were identified using custom‐written software (SparkAN; Dr A. Bonev, University of Vermont, Burlington, VT), which detects temporally delineated increases in fractional fluorescence (*F*) greater than 0.26 above the baseline fluorescence level (*F*_o_) in a defined, 1.1 × 1.1 *µ*m area of interest (5 × 5 pixels). We used this threshold amplitude because it is reliably away from the noise of about three times the standard deviation of the baseline. Noise differs from record to record and also depends on the selection box size. We chose the value of 1.26 F/Fo in order to avoid false positives from all of our records. We have published in Physiological Reports previously using this threshold (Baylie et al. 2017) and also in another publication (Khavandi et al. 2016). Increases in Ca^2+^ concentration ([Ca^2+^]) are expressed as *F*/*F*_o_. At least two areas from each artery were analyzed for each experiment and the average was used as *n* = 1 for analysis.

#### Measurement of caffeine‐induced Ca^2+^ transients

Mesenteric arteries were loaded with Fluo‐4‐AM, mounted in a pressure myograph, pressurized to 20 or 110 mmHg and imaged with a spinning‐disc confocal microscope as described above. To prevent contraction and thus loss of focus, the arteries were incubated with 1 *μ*mol/L wortmannin, which does not affect caffeine‐induced [Ca^2+^] transients in myocytes. Arteries were exposed to caffeine (10 mmol/L) for ~5 sec (bolus applied directly to chamber with artery with continuously circulating bath solution during a 2‐min recording period and caffeine‐induced [Ca^2+^] transients imaged at 10 Hz with a 20‐msec exposure through a 20 × water‐dipping objective (NA, 1.0). In our previous study, the caffeine‐induced [Ca^2+^] transients were imaged at 1 Hz (Khavandi et al. 2016). SparkAN software was used to calculate the change in *F*/*F*_0_, for the entire field of view, obtained by dividing the fluorescence in a region of interest (ROI) by the average fluorescence of 10 preceding images from the same ROI without activity. The area under the curve for each of the caffeine‐induced [Ca^2+^] transients was calculated using a trapezoidal numerical integration of (*F*−*F*_0_)/*F*_0_ over time. The caffeine‐induced Ca^2+^ transient was considered as any change in F/Fo from the precaffeine baseline.

### Wire myography

Isolated second‐order mesenteric arteries from WT C57BL/6j and PKG[C42S]^KI^ mice were mounted in a tension myograph (Danish Myo Technology A/S, Aarhus, Denmark) and stretched to the optimal resting tension using the normalization software module. Arteries were equilibrated in oxygenated (95% air:5% CO_2_) PSS at 37°C, prior to testing vitality with 60 mmol/L K^+^‐enriched physiological saline. Vessels were then incubated with vehicle or 1 *μ*mol/L paxilline for 15 min to block BK channels, before adding 3 *μ*mol/L U46619 to induce tension. This was followed by cumulatively increasing concentrations of H_2_O_2_ (10–300 *μ*mol/L).

### Statistics

Statistical analyses were performed with GraphPad software by the Student's *t*‐test or multiple points ANOVA.* P* *<* 0.05 was considered statistically significant.

## Results

### Intraluminal pressure and calcium sparks: a “step and a ceiling”

**Figure 1:**
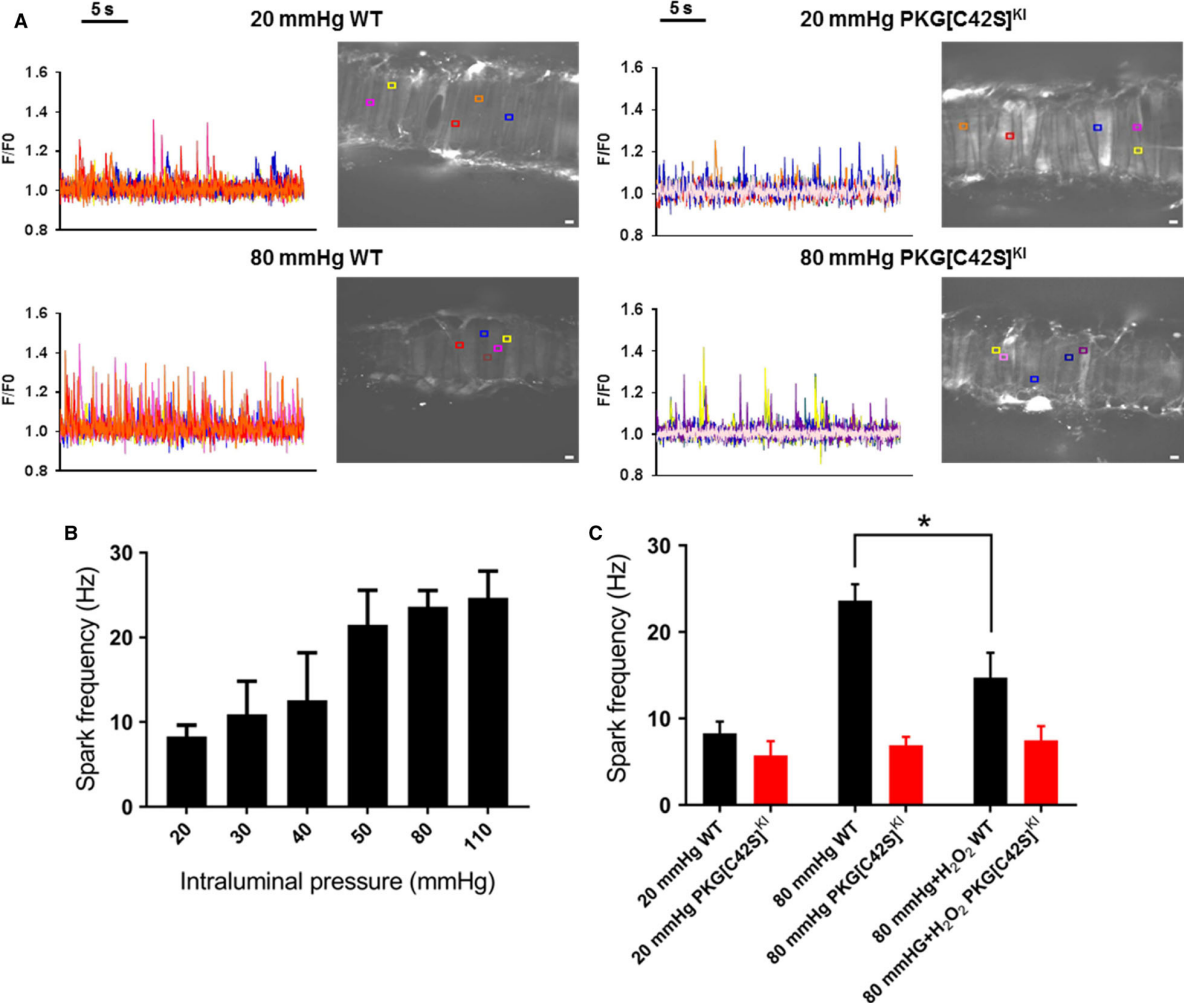
“Digital” activation mode of oxidant–PKG–Ca^2+^ spark system. (A) Ca^2+^ spark activity in representative mesenteric arteries isolated from WT (left) and PKG[C42S]^KI^ (right) mice and pressurized at either 20 or 80 mmHg. Colored lines indicate changes in fractional fluorescence (F/F_0_) at individual ROIs corresponding to the boxes of the same color shown in the artery image. White scale bar = 5 *μ*m. (B) Ca^2+^ spark frequency in WT arteries pressurized at 20, 50, 80, or 110 mmHg (*n* > 8 arteries from >6 mice) or (C) PKG[C42S]^KI^ arteries at 20 or 80 mmHg (*n* = 9 arteries from 3 mice). Data are means ± SEM.

We first studied the relationship between intraluminal pressure and Ca^2+^ spark frequency in third order mesenteric resistance arteries from WT (C57Bl6/j background) mice. Ca^2+^ sparks were imaged in arteries at the following incremental intraluminal pressures: 20, 30, 4, 50, 80, and 110 mmHg. Examples of Ca^2+^ spark recordings at 20 and 80 mmHg are shown in Figure 1A. As described previously (Jaggar 2001), there was a rise in Ca^2+^ spark frequency with increasing pressure, but two potentially notable findings were apparent. First, there was a graded “pressure‐step” between 20 and 50 mmHg and second, once Ca^2+^ spark frequency had risen to 50 mmHg there was no further increase in frequency at higher intraluminal pressures; that is, there was a “ceiling effect” regarding the generation of Ca^2+^ sparks through pressure (Fig. 1B). Ca^2+^ sparks were also recorded from arteries of the PKG[C42S]^KI^ mouse at low and high pressures (Fig. 1C). Consistent with our earlier observation, Ca^2+^ spark frequency at low pressures was equivalent to that seen in WT arteries, but no rise in spark frequency was seen at higher intraluminal pressure (Fig. 1C).

### Exogenous oxidants have no additive effect on Ca^2+^ spark frequency

We have previously shown that incubation of arteries at low pressure with H_2_O_2_ increases Ca^2+^ spark frequency similarly to that triggered by raising intraluminal pressure (Khavandi et al. 2016). Here we sought to determine whether PKG, once activated by raised intraluminal pressure, could be further activated by exogenous oxidants and thus drive additional Ca^2+^ spark formation and vasodilation. When WT arteries pressurized at 80 mmHg of intraluminal pressure were incubated with 30 *µ*mol/L H_2_O_2_ there was in fact a significant reduction in Ca^2+^ spark frequency (Fig. 1C, Baseline Hz: 23.5 ± 1.9 vs. H_2_O_2_ Hz: 13.3 ± 2.8, *P* = 0.04) possibly representing hyperpolarization of the artery and subsequently a reduction in Ca^2+^ entry through voltage‐gated Ca^2+^ channels. However, this observation also suggests that once the pressure–oxidant–PKG axis is active and stimulating Ca^2+^ spark formation, it may not be possible to further boost this pathway using exogenously applied oxidants. We attempted to measure Ca^2+^ sparks in pressurized arteries by depolarizing the VSMC membrane potential through application of high K^+^ solution, as previously demonstrated in middle cerebral arteries (Jaggar 2001). However, in the mesenteric resistance arteries, application of either 30 or 60 mmol/L KCl immediately initiated a profound global influx of Ca^2+^ so that any Ca^2+^ spark activity was obscured.

### Oxidant‐activated PKG requires intraluminal pressures to trigger BK vasodilation

Pressure myography and wire myography are the two principal techniques used currently to study ex vivo small artery contractility. These two methods differ significantly in their mechanical approach to the artery under study. Thus, while pressure myography initiates spontaneous myogenic constriction secondary to the intraluminal pressure, wire myography stretches the artery using two wires threaded through the lumen and constriction is induced using external agonists (e.g., U46619). From a physiological perspective, the difference between these approaches is potentially important as we previously showed that it is specifically the rise in intraluminal pressure which triggers oxidant activation of PKG and thus Ca^2+^ spark vasoregulation (Khavandi et al. 2016). Therefore, we compared the vasodilation to H_2_O_2_, which activates the oxidant–PKG–Ca^2+^ spark pathway, between WT and PKG[C42S]^KI^ arteries when measured using wire or pressure myography.

**Figure 2:**
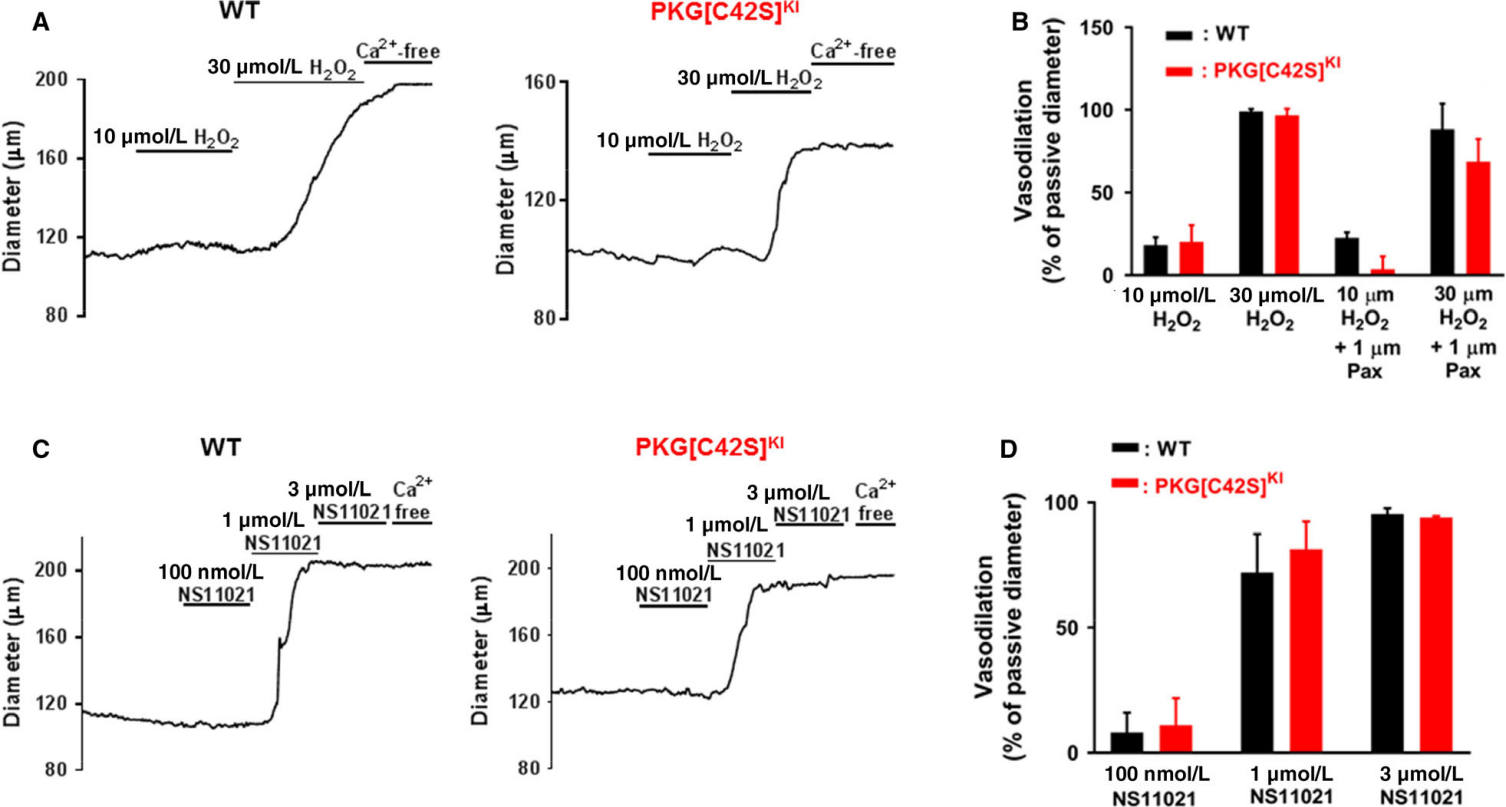
Functional vasodilatory capacity of oxidant‐activated PKG*.* (A) Representative records of H_2_O_2_‐induced vasodilation of WT (left) and PKG[C42S]^KI^ (right) arteries pressurized to 80 mmHg. (B) Changes in artery diameter induced by 10 and 30 *µ*mol/L H_2_O_2_ in the absence or presence (pax) of 1 *µ*mol/L paxilline, expressed as percent of the difference between the maximal passive diameter in the absence of extracellular Ca^2+^ (maximal dilation = 100%) and the initial diameter. Bars represent mean ± SEM of *n* = 7 arteries from five mice (WT control), *n* = 7 arteries from six mice (PKG[C42S]^KI^ control), *n* = 4 arteries from four mice (WT paxilline) or *n* = 3 arteries from three mice (PKG[C42S]^KI^ paxilline). (C) Records of vasodilation induced by NS11021 (0.1–3 *µ*mol/L) in arteries from PKG[C42S]^KI^ (left, *n* = 4) and WT (right, *n* = 4) mice at 80 mmHg. (D) Changes in artery diameter induced by NS11021. Bars represent mean ± SEM.

Figure 2A illustrates the H_2_O_2_‐induced vasodilation of arteries at an intraluminal pressure of 80 mmHg studied using pressure myography. Intriguingly, there was no difference in the vasodilation to H_2_O_2_ between the arteries from WT or PKG[C42S]^KI^ mice (Fig. 2B). Given that the sole difference between WT and PKG[C42S]^KI^ mesenteric arteries is the capacity to activate PKG through oxidation, equivalence of the H_2_O_2_ vasodilation between the two groups suggests that the vasodilatory effect of the applied H_2_O_2_ is independent of BK channel recruitment via the oxidant–PKG–Ca^2+^ spark pathway. Consistent with this observation, the vasodilation of the pressure‐constricted arteries to H_2_O_2_ was unchanged after incubation with the BK channel blocker, paxilline (Fig. 2B – vasodilation presented as a percentage of passive diameter), applied to prevent hyperpolarization arising from activation of the PKG–Ca^2+^ spark–BK channel pathway.

We also examined the influence of the intraluminal pressure–oxidant–PKG–Ca^2+^ spark pathway on the functional availability of the resistance artery BK channel population in the setting of pressure‐induced constriction. BK channel vasodilation by Ca^2+^ sparks is the final physiological step of the pressure‐induced oxidative pathway. However, the contribution of BK channel vasodilation to overall tone is small, perhaps accounting for only around 25% reduction in the overall inherent pressure‐induced constriction of WT arteries (and around 10% of the “passive” diameter seen in Ca^2+^‐free solution). In PKG[C42S]^KI^ arteries an increase in pressure causes constriction that is not counteracted by activation of BK channels, as evidenced by a lack of effect of paxilline on artery diameter (Khavandi et al. 2016). However, it is not established whether the absence of the oxidant‐driven PKG pathway interferes with the vasodilatory function of the BK channel population as a whole. In order to examine this, we measured vasodilation of pressure‐constricted WT and PKG[C42S]^KI^ arteries in response to the BK channel agonist NS11021. Figure 2C shows original records of NS11021‐induced dilation, from which the mean amplitudes of responses to 0.1, 1, and 3 *μ*mol/L NS11021 were measured. The results show that vasodilation to NS11021 at all three concentrations was identical in WT and PKG[C42S]^KI^ arteries (Fig. 2D).

**Figure 3:**
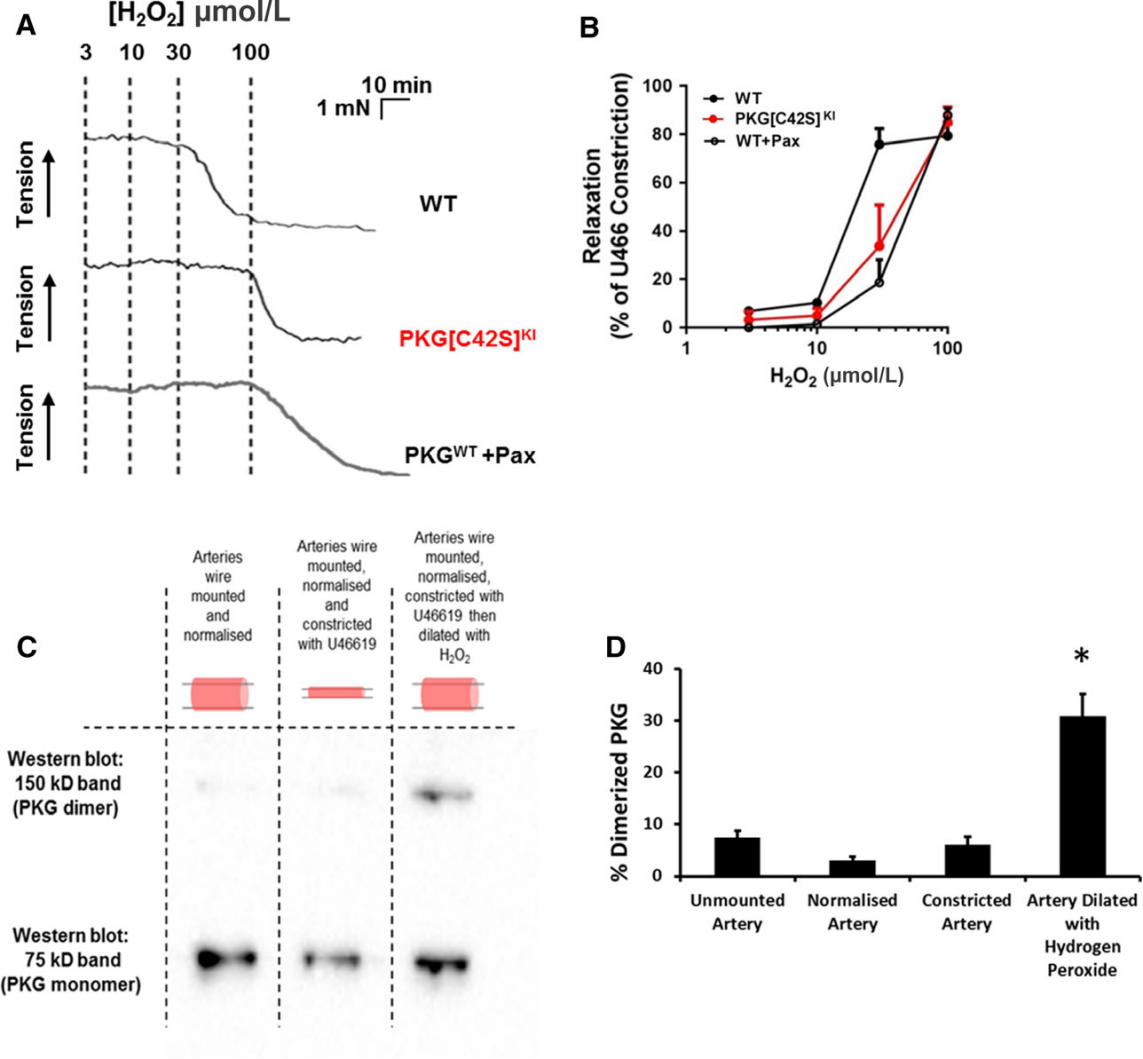
Effects of H_2_O_2_ on wire‐mounted mesenteric arteries. (A) Representative records of H_2_O_2_‐induced vasodilation of WT (with or without Paxilline) and PKG[C42S]^KI^ arteries mounted in a wire myograph and constricted with 3 *μ*mol/L U46619. (B) Concentration–response relationships for H_2_O_2_‐induced vasodilation of WT (*n* = 7 arteries from five mice) and PKG[C42S]^KI^ (*n* = 7 arteries from four mice) arteries, as well as WT arteries in the presence of 1 *μ*mol/L paxilline (*n* = 4 arteries from three mice). Data are means ± SEM. Relaxation is expressed as percent of the maximal constriction evoked by U46619. (C) Western blot of protein lysates extracted from wire‐mounted arteries, showing bands recognized by the PKG antibody at 75 kD (PKG monomer) and 150 kD (PKG dimer). Lysates were extracted from arteries stretched using the normalization protocol, with and without the addition of 3 *μ*mol/L U46619 or U46619 followed by 100 *μ*mol/L H_2_O_2_. (D) Bar graph comparing relative PKG dimerization in the different conditions (* indicates *P* < 0.01 for difference in the percentage of dimerized PKG in group of arteries normalized, constricted, and then dilated with H_2_O_2_ (*n* = 4) compared with all three other groups respectively: unmounted arteries (*n* = 6), normalized arteries (*n* = 4) and constricted arteries (*n* = 6)).

The effects of H_2_O_2_ on mesenteric arteries were markedly different when using wire rather than pressure myography. This wire‐based protocol has previously been published by Prysyazhna et al. (2012) and our findings are consistent with theirs; namely a rightward shift in the vasodilatory potency of H_2_O_2_ in PKG[C42S]^KI^ compared with WT arteries (Fig. 3A and B). However, as can be seen from Figure 3A, vasodilation to H_2_O_2_ occurred over only two concentrations applied, so the capacity to generate ED50 values was suboptimal. Nevertheless, in WT arteries, H_2_O_2_ produced concentration‐dependent relaxation of WT mouse artery with EC_50_ = 17.3 ± 1 *µ*mol/L (*n* = 7), pEC_50_ = 4.77 ± 0.03 and maximal response above 30 *µ*mol/L. When tested on arteries from PKG[C42S]^KI^ mice, or WT arteries in the presence of paxilline, H_2_O_2_ at 30 *µ*mol/L had reduced effect on force and furthermore the induced responses often failed to reach a clear maximum at 100 *µ*mol/L, the highest concentration tested, and this further impeded analysis. Accurate EC_50_ values could not, therefore, be determined for each preparation in these conditions. Comparison of the mean concentration–response curves generated in each condition (Fig. 3B), by repeated measures two‐way ANOVA followed by Tukey’s multiple comparisons test, indicated a significant loss of H_2_O_2_ potency in WT arteries when they were treated with paxilline (*P* = 0.0034). The H_2_O_2_ concentration–relaxation relationship measured in arteries from PKG[C42S]^KI^ mice, previously described by Prysyazhna and Eaton (Prysyazhna et al. 2012), closely resembles that from WT arteries exposed to paxilline. The concentration–response curves in each condition were fitted with the following equation:

$$R=R_{\min}+\;\left(R_{\max}-R_{\min}\right)/\left(1+{\left(X/\text{EC}50\right)}^{-n}\right).$$

where *R* represents the relaxation at *X* concentration of H_2_O_2_, *R*_min_ and *R*_max_ are the minimum and maximum responses at zero and high H_2_O_2_ concentrations, respectively, and n is a slope factor describing the steepness of the relationship. The best fit curves gave EC_50_ values of 58 *µ*mol/L (pEC_50_ = 4.2) for paxilline‐treated arteries from WT mice and 37 *µ*mol/L (pEC_50_ = 4.4) for arteries from KI mice (Fig. 3B). There was therefore a two‐ to threefold loss of H_2_O_2_ potency when BK_Ca_ channels were blocked or PKG oxidation prevented.

Increasing intraluminal pressure within small mesenteric resistance arteries initiates dimerization of PKG independently of exogenously applied H_2_O_2_ (Khavandi et al. 2016). The comparison of the induced vasodilation between wire and pressure myography suggests that the stretch and agonist‐induced constriction of resistance arteries during wire myography may not activate PKG through dimerization. To investigate this possibility, we measured PKG dimerization in individual arteries mounted in a wire myograph, using western blot protocols. Measurements were made on arteries that were: (i) stretched via normalization, (ii) constricted with U46619, and (iii) constricted with U46619 then dilated with H_2_O_2_. Figure 3C and D show that a significant increase in PKG oxidative dimerization only occurred following the application of H_2_O_2_. Neither the initial stretch of the artery during normalization nor the constriction induced with U46619 caused PKG dimerization. Additional examples of western blot protocols from wire mounted arteries are shown as a Figure S1.

### Oxidant activation of PKG modulates SR Ca^2+^ release rather than loading

Until recently, evidence pointed to a mechanism whereby intraluminal pressure within resistance arteries triggers the rise in Ca^2+^ spark frequency via the depolarization of VSMCs as the artery constricts (Knot and Nelson 1995; Knot and Nelson 1998; Jaggar et al. 1998). It was thought that the resulting Ca^2+^ influx through voltage‐dependent Ca^2+^ channels then “loads” the SR via SERCA. The failure of this process in PKG[C42S]^KI^ arteries suggests an additional layer of complexity within the VSMC and indicates that oxidant‐activated PKG must be an intermediary between intraluminal pressure and the SR. There are two possible routes by which oxidized PKG could regulate Ca^2+^ spark frequency: Stimulation of SR loading with Ca^2+^ or regulation of the release of Ca^2+^ sparks from the SR.

To elucidate the effects of oxidized PKG on the SR in resistance arteries, we studied the Ca^2+^ transient induced by the rapid application of a 10 mmol/L caffeine bolus. At this concentration, caffeine opens RyR and the amplitude of the resulting Ca^2+^ transient, seen as a wave of Ca^2+^ through the cell, is accepted as an accurate surrogate for the measurement of SR Ca^2+^ content (Liang et al. 2012). In our previous study of oxidant‐activated PKG in resistance arteries (Khavandi et al. 2016), we had imaged the caffeine‐induced [Ca^2+^] transient at 1 Hz, similar to previously published approaches (Liang et al. 2012). Using this approach, the amplitude appeared to be greater in the PKG[C42S]^KI^ arteries but this difference between the WT and PKG[C42S]^KI^ arteries was not significant. Therefore, for this study we used a more rapid imaging frequency to assess the caffeine‐induced [Ca^2+^] transient (10 Hz) in order to magnify any differences between the WT and PKG[C42S]^KI^ arteries and also calculated the Area under the curve (AUC) and the time‐to‐ peak. Furthermore, in this study the caffeine‐induced Ca^2+^ transient was measured in intact arteries at intraluminal pressures of 20 mmHg, at which point the PKG–Ca^2+^ spark pathway is dormant, and at 110 mmHg which is an intraluminal pressure at which, Ca^2+^ sparks in WT vessels are present.

**Figure 4:**
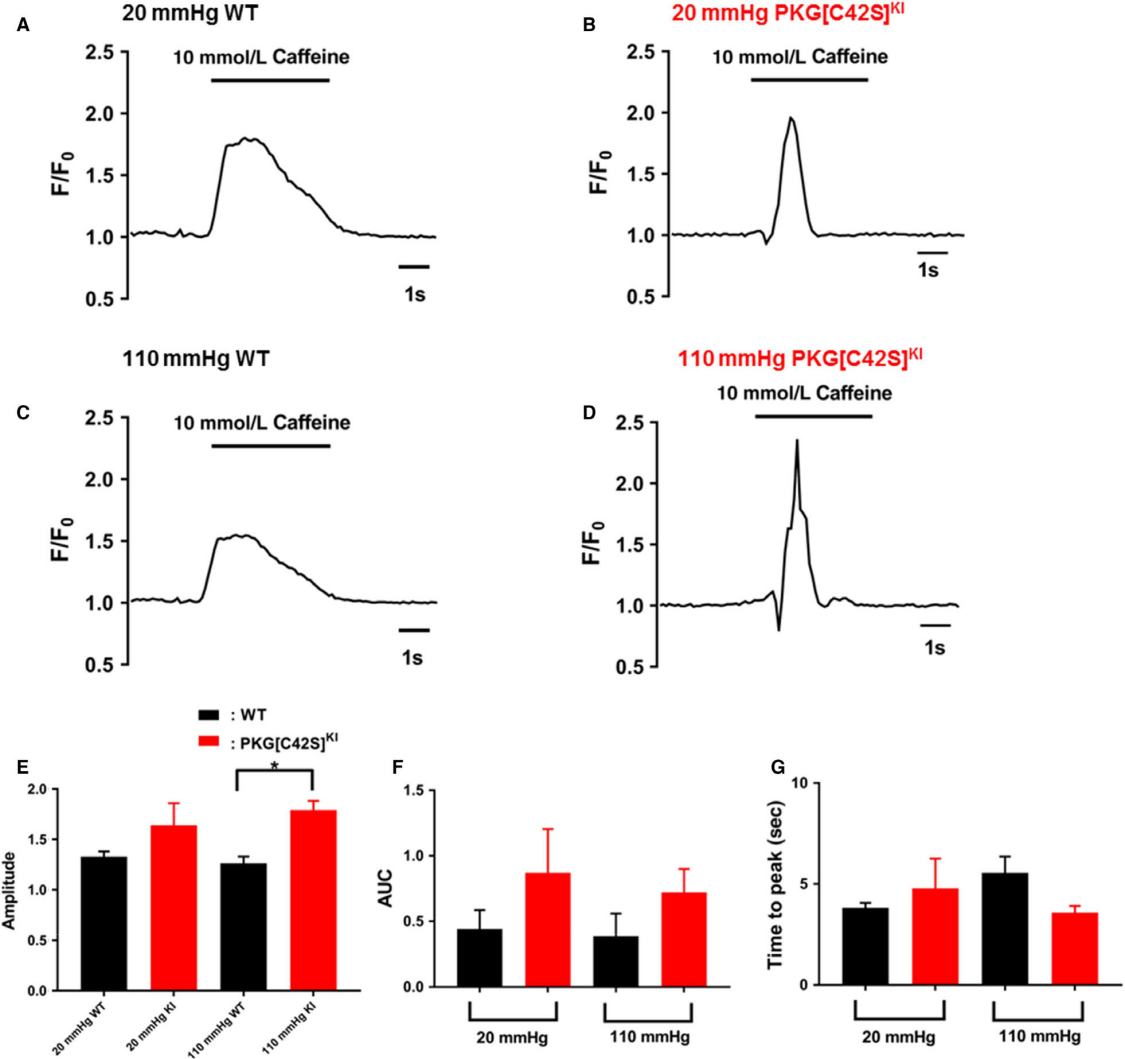
Caffeine‐induced [Ca^2+^] transients are decreased in WT arteries. (A–D) Representative traces of changes in fractional fluorescence (*F*/*F*_0_) following the application of 10 mmol/L caffeine to arteries from WT or PKG[C42S]^KI^ mice at either low or high intraluminal pressures. (E–G) Comparison of the Area Under the Curve (AUC), Time to Peak or peak amplitude (*F*/*F*_0_) of responses to caffeine between WT (*n* = 7 arteries from four mice) and PKG[C42S]^KI^ (*n* = 5 arteries from four mice) arteries at intraluminal pressures of 20 and 110 mmHg. *P < 0.001 between WT and PKG[C42S]KI arteries at 110 mmHg for *F*/*F*_0_.

We hypothesized that any interruption to Ca^2+^ loading of the SR would be associated with an overall reduction in the caffeine‐induced Ca^2+^ transient. Conversely, if Ca^2+^ release were to be disabled, then the SR Ca^2+^ content would not be affected. Examples of Ca^2+^ transients induced by rapid application of a caffeine bolus at low (20 mmHg) and high (110 mmHg) intraluminal pressures are shown in Figure 4A–D for WT and PKG[C42S]^KI^ arteries. As summarized in Figure 4E–G, at low intraluminal pressure there was no difference in the Area under the Curve (AUC), amplitude (as determined by the rise from baseline of the *F*/*F*_0_) or the time ‐to‐peak of the caffeine‐induced Ca^2+^ transient between the WT and PKG[C42S]^KI^ arteries. At high intraluminal pressure (110 mmHg), although the AUC and time‐to‐peak were equivalent between groups, the amplitude of the caffeine‐induced [Ca^2+^] transient was significantly higher in arteries from PKG[C42S]^KI^ mice than from WT mice. Raising intraluminal pressure did not affect AUC, amplitude, or the duration of the caffeine‐induced Ca^2+^ transient within groups (WT, PKG[C42S]^KI^ arteries).

## Discussion

The principal findings from this study illustrate a distinctive activation step and a functional “ceiling” of the vasodilatory pathway within resistance arteries triggered by intraluminal pressure: oxidant generation–PKG–Ca^2+^ sparks–BK channel vasodilation. We show the importance of intraluminal pressure specifically as the prompt to activate the oxidant‐driven pathway and illustrate the functional independence of BK channel bioavailability from oxidant‐activated PKG in arteries with steady state constriction. Our data suggest that once oxidized and activated, PKG regulates Ca^2+^ spark release from the SR rather than influencing SR Ca^2+^ loading. We propose that these distinctive characteristics enable resistance arteries not only to maintain the requisite degree of BK channel vasodilation to regulate pressure‐induced constriction of the artery but also to prevent excessive vasodilation which would disturb local flow.

Ca^2+^ spark vasoregulation of small arteries is one of the most important determinants of contractile tone and thus microvascular function (Wellman and Nelson 2003). Each Ca^2+^ spark activates around 30 neighboring BK channels in the plasma membrane and the resultant hyperpolarization provides a counterbalance to the pressure‐induced constriction (Perez et al. 2001). The balance of these two processes (Ca^2+^ spark–BK vasodilation vs. pressure‐induced constriction) is weighted heavily in favor of the contractile mechanism. Thus, at steady state pressure‐induced constriction, inhibiting BK channel function further constricts a mesenteric artery by an additional 10% of the overall passive diameter. This is particularly important for conditions such as obesity (Nystoriak et al. 2014) or type 2 diabetes (Mokelke et al. 2003; 2005), where BK channel function is impaired and the increase in resistance artery constriction contributes to the development of hypertension. The reduction in BK channel function in these metabolic disorders is often attributed to reduction in Ca^2+^ sensitivity of the channel(Rusch 2009; Nystoriak et al. 2014), but in one study, a reduction in Ca^2+^ spark frequency has also been observed (Mokelke et al. 2005). As such, elucidation of the mechanisms underpinning the coupling between Ca^2+^ sparks and BK channels could lead to future therapeutic opportunities in the field of hypertension.

In this regard, we recently demonstrated a previously unsuspected mechanism by which oxidants generated within the VSMC of small arteries in response to intraluminal pressure regulate Ca^2+^ spark generation through the activation of protein kinase G (PKG) (Khavandi et al. 2016). We demonstrated this pathway through the use of a genetic “knock‐in” mouse in whom the capacity of PKG to be activated by oxidants is disabled through a single amino acid substitution from cysteine at point 42 to a serine (PKG[C42S]^KI^) mouse (Prysyazhna et al. 2012). From a cardiovascular perspective, the PKG[C42S]^KI^ mouse model is notable as it has hypertension (Prysyazhna et al. 2012). We found that the increase in blood pressure in the PKG[C42S]^KI^ mouse was accounted for by reduction in intraluminal pressure‐induced Ca^2+^ spark frequency in the VSMC, preventing functional activation of the BK channels (Khavandi et al. 2016). This pathway: intraluminal pressure–oxidant formation–PKG activation–Ca^2+^ spark–BK channel vasodilation is therefore constitutively active during pressure‐induced constriction of small arteries. However, questions remained unanswered regarding the mechanical stimulus threshold necessary to activate and maintain this functional architecture and how oxidant‐activated PKG engages with the VSMC sarcoplasmic reticulum in order to maintain Ca^2+^ spark frequency.

In this study, we observed a graded step activation pattern for the pressure‐induced oxidant‐mediated increase in Ca^2+^ spark frequency. The physiological trigger for pathway activation appeared to be between 20 mmHg and 50 mmHg of intraluminal pressure. Furthermore, when intraluminal pressure was elevated beyond 50 mmHg or H_2_O_2_ was applied to pressurized arteries, there was no further increase in Ca^2+^ spark frequency. Hence in the text, we refer to this phenomenon as a “functional ceiling”. Given that resistance arteries of this size almost continuously experience a level of intraluminal pressure between 50 and 100 mmHg, we suggest that in vivo, the oxidant–PKG–Ca^2+^ spark pathway is not only continuously active, but is fully activated. Consistent with this concept, pressure‐constricted arteries from both WT and PKG[C42S]^KI^ mice vasodilated equivalently to external H_2_O_2_, both in the presence and absence of paxilline, indicating that this dilation occurred independently of BK channels.

From a physiological perspective, there are benefits to an oxidant‐driven vasoregulatory system with this distinctive steep dependence on pressure and functional ceiling configuration. First, when considering the vascular smooth muscle cell layer of resistance arteries, the primary function is to provide a steady baseline of the contractile tone, which can be rapidly modulated to meet local metabolic demand, predominantly through endothelial or perivascular mechanisms. However, following this externally induced vasodilation, the VSMC layer must return to steady‐state pressure‐induced tone. The functional conformation of oxidant‐activated PKG is well suited to this function in that the pathway remains almost permanently active and thus maintains Ca^2+^ spark frequency at the level required to maintain BK channel activation and balance the inherent pressure‐induced constriction. In this configuration, the resistance artery BK channel population is submaximally active, as evidenced by the capacity of the BK channel agonist NS11021 to further vasodilate the artery. In addition, the identical vasodilatory responses to NS11021 of the WT and PKG[C42S]^KI^ arteries suggest that BK channels are not directly regulated by oxidant‐activated PKG. From a pathophysiological perspective, overproduction of oxidants is a feature of many cardiovascular pathologies (Al Ghouleh et al. 2011; Nguyen Dinh Cat et al. 2013). In this context, the functional ceiling of the pressure–oxidant–PKG–Ca^2+^ spark pathway would be beneficial as excessive oxidant production would be prevented from initiating potentially disruptive additional BK channel vasodilation.

To further investigate the interaction of oxidant‐activated PKG with the SR, we studied caffeine‐induced Ca^2+^ transients in intact arteries from WT and PKG[C42S]^KI^ arteries at both low (20 mmHg) and elevated (110 mmHg) intraluminal pressures, similar to our measurement of Ca^2+^ sparks. Compared with WT arteries, at the higher intraluminal pressure, the lower Ca^2+^ spark frequency of the PKG[C42S]^KI^ arteries was associated with a higher caffeine‐induced Ca^2+^ transient amplitude. Importantly, however, there were no differences in the “Area Under the Curve” (AUC) or “Time to Peak” for the caffeine‐induced Ca^2+^ transients; and in this context, the AUC is probably a more accurate interpretation of the overall SR Ca^2+^ load than the peak amplitude. Our interpretation of these divergent differences (fewer Ca^2+^ sparks in physiologically pressurized PKG[C42S]^KI^ arteries in conjunction with higher amplitudes of the Ca^2+^ transient but equivalent AUC) is that whilst Ca^2+^ loading of the SR is maintained in the PKG[C42S]^KI^ arteries, Ca^2+^ release may be impeded. These observations contrast to those observed in isolated cardiomyoctes, where a failure of of phospholamban phosphorylation in PKG[C42S]^KI^ mice is associated with a smaller caffeine‐induced Ca^2+^ transient (Scotcher et al. 2016). We saw almost the opposite, albeit in intact arteries rather than isolated cardiac myocytes and we propose that in contrast to the heart, oxidant‐activated PKG within arteries regulates Ca^2+^ release from the SR rather than Ca^2+^ loading, possibly due to an increase in the number of RyRs. Alternatively, there may be differences in SR Ca^2+^ handling mechanisms in the PKG[C42S]^KI^, for example SERCA pump activity. However, it is interesting to note that serine residue at position 2808 of RyR has previously been identified as a target for PKG within a kinase phosphorylation “hotspot” (Camors and Valdivia 2014; Ho et al. 2016) and further vascular studies are warranted in this regard.

Our data also illustrate the importance of intraluminal pressure as a specific trigger to activate the oxidant–PKG–Ca^2+^ spark vasodilatory pathway. Wire and pressure myography exert very different mechanical forces across the arterial wall which in turn results in different responsiveness to H_2_O_2_. Thus, exogenous H_2_O_2_ had equivalent effects on WT and PKG[C42S]^KI^ arteries with spontaneous myogenic constriction studied in the pressure myograph. Conversely, applied to wire‐mounted preconstricted arteries, equivalent concentrations of H_2_O_2_ showed a twofold greater vasodilatory potency on WT arteries compared with PKG[C42S]^KI^ arteries, consistent with an earlier study (Prysyazhna et al. 2012). Our interpretation of this difference between the two approaches to vascular study is that pressure myography activates the oxidant–PKG–Ca^2+^ spark pathway but wire myography does not. Thus, in pressure‐constricted arteries where PKG is already activated by oxidants, there can be no further vasodilation through this mechanism (the “functional ceiling” effect). However, in wire‐mounted arteries, exogenous H_2_O_2_ is required to activate PKG and this accounts for the impaired vasodilation to H_2_O_2_ seen in the PKG[C42S]^KI^ arteries compared with WT. This interpretation was reinforced by the western blot protocols which indicated that to induce oxidative dimerization of PKG within wire‐mounted arteries, it was necessary to incubate with H_2_O_2_. Whether this difference between the techniques reflects a failure of stretch from the transluminal wire to generate oxidants, or perhaps wire‐induced disruption of the myocyte microarchitecture required to anchor PKG adjacent to oxidant producers or the SR, is an avenue for further study. Nevertheless, while this is perhaps a subtle difference between the approaches (wire vs. pressure myography) and will not be relevant for the majority of vascular studies, our observation here has ramifications for the choice of technique used to study Ca^2+^ sparks or BK channel vasodilatory function in resistance arteries. A caveat is that wire myography can only be applied to arteries around twice the intraluminal diameter (200–400 *µ*m) of those used for pressure myography. As pressure‐induced constriction is generally seen in arteries of smaller diameter (less than 200 *µ*m), the observed difference between wire and pressure myography could reflect heterogeneity due to size.

Also left unanswered is the question of how intraluminal pressure generates oxidants within small arteries to regulate Ca^2+^ spark vasodilation. In cardiomyocytes, a microtubule network detects mechanical stretch and facilitates activation of NADPH Oxidase 2 (Nox2) to generate oxidants which regulate Ca^2+^ spark frequency (Prosser et al. 2011). It is established that intraluminal pressure within arteries from such diverse circulatory beds as mesenteric (Khavandi et al. 2016), skeletal (Nowicki et al. 2001), and renal (Ren et al. 2010) territories generates oxidants. It would be intriguing if microtubules and Nox2 play similar roles in the vasculature as demonstrated in the heart as this would represent a previously unsuspected oxidant pathway in the regulation of small artery diameter.

In summary, our work clarifies a mechanism that lies at the heart of small artery autoregulation; specifically, the processes underpinning the Ca^2+^ spark–BK channel vasodilatory buffer, which balances against inherent pressure‐induced constriction. The initiation of Ca^2+^ sparks, via pressure‐induced oxidants that activate PKG, is triggered at an intraluminal pressure threshold between 20 and 50 mmHg, but once the system is activated neither pressure nor oxidants can further increase Ca^2+^ spark frequency or vasodilation through this mechanism. We suggest that this feature of pressure‐induced vasodilation is crucial for normal microvascular diameter regulation. The principal role of the VSMC layer of resistance arteries is to provide a steady basal blood flow to distal beds, which can be both quickly and easily modulated, for example by the endothelium, but then reverts back to the original steady state. The characteristics of the pressure–oxidant–PKG–Ca^2+^ spark pathway, a steep sensitivity to intraluminal pressure and functional ceiling, suit this role well. Our work also suggests that oxidant‐activated PKG regulates Ca^2+^ spark release rather than Ca^2+^ loading of the SR, but further research is required to elucidate the microdomain that is likely to accommodate the pressure‐sensitive oxidant generators, PKG and the SR.

## Conflict of Interest

The authors declare no conflict of interest.

## Supporting information




**Figure S1.** Representative Western blots illustrating presence of significant dimerization of PKG in wire‐mounted arteries only following incubation/vasodilation with H_2_O_2_.
