# Supplementary material for: “A Step and a Ceiling”: mechanical properties of Ca2+ spark vasoregulation in resistance arteries by pressure‐induced oxidative activation of PKG
Source: Physiol Rep. 2019 Nov 28;7(22):e14260. doi: 10.14814/phy2.14260 (PMC6883097; doi:10.14814/phy2.14260)
Supplement: Supplementary file 1 — Figure S1. Representative Western blots illustrating presence of significant dimerization of PKG in wire‐mounted arteries only following incubation/vasodilation with H2O2. [file PHY2-7-e14260-s001.docx]

Supplementary Figure

**UNMOUNTED**

**ARTERT**

**NORMALISED ARTERT**

**CONSTRICTEDARTERY (U46619)**

**DILATED**

**ARTERY**

**(H_2_O_2_)**


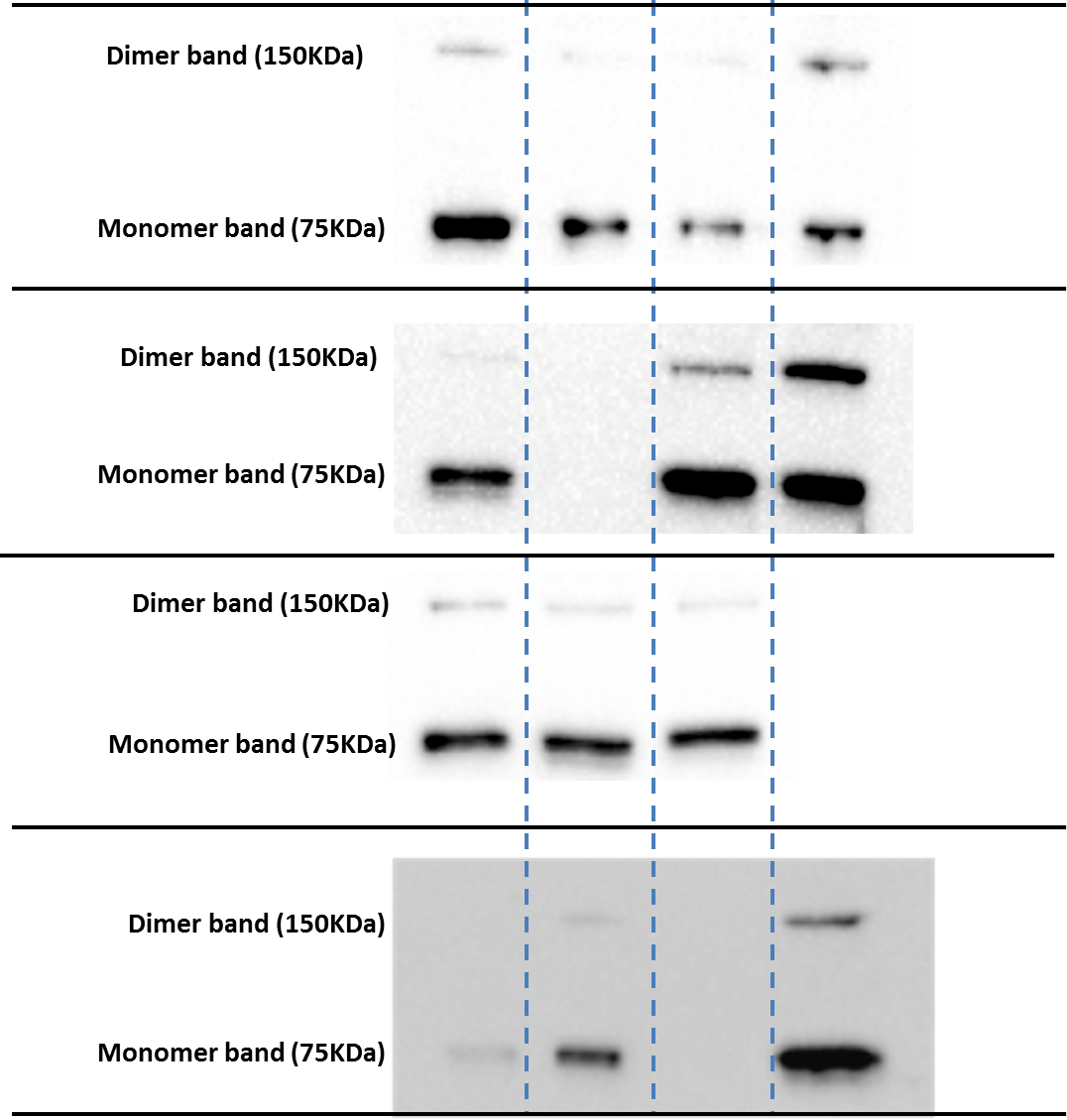


Supplementary Figure 1

Representative Western blots illustrating presence of significant dimerization of PKG in wire mounted arteries only following incubation/vasodilation with H_2_O_2_
